# A Case of Dysmenorrhœa and Leucorrhœa Produced by Ante-Flexion of the Uterus, Accompanied by Endo-Cervicitis

**Published:** 1873-06

**Authors:** J. T. Jelks

**Affiliations:** Marietta, Georgia


### A CASE OF DYSMENORRHŒA AND LEUCORRHŒA PRODUCED BY ANTE-FLEXION OF THE UTERUS, ACCOMPANIED BY ENDO-CERVICITIS.

BY J. T. JELKS, M.D., MARIETTA, GEORGIA.

Mrs. G. N., of Jasper county, consulted me for dysmenorrhœa, leucorrhœa, and resulting sterility.

She is about twenty-two years of age, weight one hundred and forty pounds, the mother of one child—the child dying during the labor, it being a breech presentation, and the head remaining in the inferior straight about two hours.

Her labor occurred about eighteen months since. It was some weeks before she could get from her bed. She has since suffered from excessive leucorrhœal discharge, and when her menses appeared they gave her very great pain—"almost equal to labor," the pains being followed by the discharge of clots. During her periods she suffers from excessive prostration, accompanied by hysterical manifestations.

Upon examination I found the os patulous, and the whole pelvic region very painful; bimanual examination increased the pain very much. There was resting on the bladder a hard movable tumor, causing her a great deal of distress in that organ, and inability to retain the urine long. Using considerable pressure, I reached the os uteri, and passing my finger along the base of the bladder, detected a very acute angle at the junction of the neck with the body of the uterus, and, passing my finger still further, I satisfied myself that I had a case of anteflexion of the uterus to deal with. By means of bimanual examination I could very readily get the uterus between my hand and finger, and found it larger than normal.

With the speculum I found the os uteri and cervical canal filled up with a thick, tenacious mucus, evidencing endo-cervicitis. This mucus being wiped and washed out, revealed an intensely congested and inflamed os and cervical canal, bleeding at touch of speculum, or on the slightest attempts to introduce the uterine probe of Sims. With various degrees of curvature I failed to get this instrument beyond the internal os, because of the very acute angle at that point.

Wrapping the probe with cotton, saturating this with pure carbolic acid, liquified by heat, I applied it to the cervical canal very thoroughly.

I then introduced Talliaferro's cloth tent, saturated with glycerine, after which I packed cotton and glycerine around the os. These produced a very free discharge of serum by setting up the process of exomosis, thereby relieving the congestion of the parts very much.

The next examination, three days subsequently, revealed much less tenderness on pressure. This time I saturated the tent with carbolic acid and carried it to the fundus; but was unable to pass the internal os with either the sound of Simpson, or the probe of Sims.

February 27th.—Her menses appeared, giving her much less pain than she has usually suffered at such times. She passed no clots, and instead of being "unwell" only two days, as has been the case for the previous fifteen months, she was sick for five days—her usual time before her pregnancy. Her prostration and nervousness were not so excessive as they had been previously.

March 10th.—I made an examination. Bimanual manipulation gave little or no pain; urethral trouble all gone, and she feels quite comfortable. Applications the same as before.

March 14th.—Found Mrs. N. quite cheerful, all symptoms improved, and leucorrhœa relieved. To-day, in addition to the carbolized cloth tent, cotton and glycerine, I poured through the speculum into the vagina about ten grains of tannin. After a short while this produced considerable uneasiness throughout the pelvic region, from its powerful constringing effect on the vagina and surrounding tissues. By using the warm douche some hours afterward her trouble was diminished; but it was about thirty-six hours before she could walk with any comfort.

March 21st.—To-day I found no tenderness nor leucorrhœal discharge. By the use of the speculum, I found the cervical canal clean; neither did the os nor canal bleed on being touched with the instruments.

After introducing a carbolical cloth tent up to the fundus, I wrapped the tannin in the cotton, saturated this with glycerine, and then introduced it through the speculum. This time the tannin gave no trouble. I directed her to remove the tent if the contractions of the uterus should become painful; otherwise to allow it to remain until the 24th, when she was expecting her menses.

March 24th.—Her menses appeared to-day; suffers no pain, no nervousness, no clots, and no prostration. Very much to her surprise, she did not feel the necessity of taking her bed, as has been the case for months. She walks as freely as ever, experiencing no inconvenience whatever.

She has now passed through this period—lasted her about five days. Once or twice she felt a little pain in the pelvic region, but was relieved almost immediately by elixir valerianate of ammonia. While the flexion remains yet, upon a recent examination I find the endo-cervicitis relieved. The dysmenorrhœa and leucorrhœa have been cured, and the uterus considerably reduced in size.

I hope, by returning to her home, she may become impregnated; and, should pregnancy take place, then I expect the flexion to be cured by the increased muscular development.

